# Automating Quality Measures for Heart Failure Using Natural Language Processing: A Descriptive Study in the Department of Veterans Affairs

**DOI:** 10.2196/medinform.9150

**Published:** 2018-01-15

**Authors:** Jennifer Hornung Garvin, Youngjun Kim, Glenn Temple Gobbel, Michael E Matheny, Andrew Redd, Bruce E Bray, Paul Heidenreich, Dan Bolton, Julia Heavirland, Natalie Kelly, Ruth Reeves, Megha Kalsy, Mary Kane Goldstein, Stephane M Meystre

**Affiliations:** ^1^ Health Information Management and Systems Division School of Health and Rehabilitation Sciences The Ohio State University Columbus, OH United States; ^2^ IDEAS 2.0 Health Services Research and Development Research Center Salt Lake City Veterans Affairs Healthcare System Department of Veterans Affairs Salt Lake City, UT United States; ^3^ Department of Biomedical Informatics School of Medicine University of Utah Salt Lake City, UT United States; ^4^ Division of Epidemiology Department of Medicine University of Utah Salt Lake City, UT United States; ^5^ Geriatric Research, Education and Clinical Center Salt Lake City Veterans Affairs Healthcare System Department of Veterans Affairs Salt Lake City, UT United States; ^6^ Translational Biomedical Informatics Center Medical University of South Carolina Charleston, SC United States; ^7^ Geriatric Research, Education and Clinical Center, Tennessee Valley Healthcare System Department of Veterans Affairs Nashville, TN United States; ^8^ Department of Biomedical Informatics School of Medicine Vanderbilt University Nashville, TN United States; ^9^ Palo Alto Geriatric Research, Education and Clinical Center, Veterans Affairs Palo Alto Health Care System Department of Veterans Affairs Stanford University Palo Alto, CA United States; ^10^ Medical Service Veterans Affairs Palo Alto Health Care System Palo Alto, CA United States; ^11^ Department of Medicine Stanford University School of Medicine Stanford, CA United States

**Keywords:** natural language processing (NLP), heart failure, left ventricular ejection fraction (EF), informatics, quality measures

## Abstract

**Background:**

We developed an accurate, stakeholder-informed, automated, natural language processing (NLP) system to measure the quality of heart failure (HF) inpatient care, and explored the potential for adoption of this system within an integrated health care system.

**Objective:**

To accurately automate a United States Department of Veterans Affairs (VA) quality measure for inpatients with HF.

**Methods:**

We automated the HF quality measure Congestive Heart Failure Inpatient Measure 19 (CHI19) that identifies whether a given patient has left ventricular ejection fraction (LVEF) <40%, and if so, whether an angiotensin-converting enzyme inhibitor or angiotensin-receptor blocker was prescribed at discharge if there were no contraindications. We used documents from 1083 unique inpatients from eight VA medical centers to develop a reference standard (RS) to train (n=314) and test (n=769) the Congestive Heart Failure Information Extraction Framework (CHIEF). We also conducted semi-structured interviews (n=15) for stakeholder feedback on implementation of the CHIEF.

**Results:**

The CHIEF classified each hospitalization in the test set with a sensitivity (SN) of 98.9% and positive predictive value of 98.7%, compared with an RS and SN of 98.5% for available External Peer Review Program assessments. Of the 1083 patients available for the NLP system, the CHIEF evaluated and classified 100% of cases. Stakeholders identified potential implementation facilitators and clinical uses of the CHIEF.

**Conclusions:**

The CHIEF provided complete data for all patients in the cohort and could potentially improve the efficiency, timeliness, and utility of HF quality measurements.

## Introduction

Heart failure (HF) is associated with substantial morbidity, mortality, and consumption of medical resources. HF affects approximately five million Americans and is the number one reason for discharge for Veterans treated within the United States Department of Veterans Affairs (VA) health care system [1,2]. The cost of HF care is high, and will remain a significant concern for the US health care system with high mortality; 50% of Medicare beneficiaries do not survive three years after an HF hospitalization [3,4].

The cost of treating HF in the United States is estimated to increase from US $31 billion in 2012 to US $70 billion by 2030 [5-7]. Despite decreased HF hospitalizations between 2001 and 2009, the presence of HF as a secondary condition in hospitalizations increased over the same period [7], with research suggesting that 55% of acute exacerbations were preventable [8]. HF was the fourth most common diagnosis for hospitalization in 2014 [9] and prevalence figures indicate that 6.6 million American adults 18 years of age or older (2.8%) have HF [10]. It is estimated that an additional 3 million adults (25% increase) will be diagnosed with HF by 2030 [3,5], and it is important to implement evidence-based, guideline-concordant care that can improve HF symptoms, prolong life, and reduce readmissions [3,6,11-15].

The VA HF quality measure known as Congestive Heart Failure Inpatient Measure 19 (CHI19) describes how often guideline-concordant medical therapy, in the form of angiotensin-converting enzyme inhibitor (ACEI) or angiotensin-receptor blocker (ARB) use, is provided for patients with left ventricular ejection fraction (LVEF) of <40% at the time of discharge, unless there are contraindications. The same information is currently collected for outpatients using the Congestive Heart Failure Outpatient Measure 7 (CHF7): HF-Outpatient Left Ventricular Failure (LVF) documented and Congestive Heart Failure Outpatient Measure 14 (CHF14): HF-Outpatient LVEF Less Than 40 on ACEI or ARB measures. The measurement of this information is used for accountability within the VA. The use of these measures provides key feedback to patients (through public reporting), providers, and local or regional areas, including the VA’s Veterans Integrated Service Networks [16,17]. The measures used by the VA are in alignment with Medicare and are reported publicly [18].

Our primary goal was to develop an efficient and accurate method of obtaining quality data by automating the CHI19 measure, as it is an accountability measure that has been widely used in the VA for many years, and currently requires time-consuming chart abstraction to determine through the External Peer Review Program (EPRP). EPRP provides peer review for the VA through an external medical professional association that abstracts the charts manually to populate a dashboard [19]. Additional HF measures abstracted by EPRP include Congestive Heart Failure Inpatient Measure 10 (CHI10) and Congestive Heart Failure Inpatient Measure 20 (CHI20). CHI10 refers to HF patients who were assessed for LVF at discharge or patients for whom such an assessment was planned, whereas CHI20 refers to patients who had LVEF <40% and were taking an ACEI or ARB before being admitted as inpatients.

Using automated methods to share data and measure quality for provider feedback and public reporting is a key goal of the incentives provided by the Centers for Medicare and Medicaid Services, so that certified electronic health records (EHRs) of “meaningful use” criteria can be attained [20]. Some quality measures that use only structured data from the EHR are relatively easy to automate. A challenge for automating the computation of CHI10, CHI19, and CHI20 is that, unlike quality measures that use only structured data [21], these measures require data regarding LVEF and contraindications to medications, which in the VA are primarily in free-text health record documents and are therefore more difficult to extract.

Prior research in informatics in VA showed that health information technology and the use of explicit conceptual models can not only contribute to increasing well-formed and well-grounded health informatics research [22], but can also facilitate evidence-based practice [23] through usability testing, good research design, and implementation methodology [24]. Importantly, prior research indicates that end-user considerations, including where and when the technology is required as well as stakeholder needs and goals, must be identified for successful implementation [25-30]. To this end, we initiated development of an automated natural language processing (NLP) system capable of efficient data capture that could meet end-user needs and generate data for other informatics applications, such that the system would be positioned for adoption and implementation by the VA or other health care organizations.

## Methods

### Setting and Context

For the system’s clinical basis, we used the American Heart Association/American College of Cardiology level 1A clinical evidence, which recommends assessing the left ventricular systolic function and use of ACEI or ARB if the ejection fraction (EF) is <40%, if there are no contraindications [6,31]. We used the VA Informatics and Computing Infrastructure [32] for NLP development and analysis of EHR patient data from the VA’s Corporate Data Warehouse (CDW) [33].

#### Patient Cohort Identification and Document and Structured Data Acquisition

We obtained a listing of EPRP abstracted cases involving HF patients discharged from eight VA medical centers. To approximate the general VA patient population, we selected facilities which in total were representative of the VA population in terms of race, ethnicity, and rurality in the fiscal year 2008 to serve as our study cohort. The patient cohort was randomized and split into training and test sets based on the sampling strategy described below. We obtained the associated text integration utilities (TIUs) notes for each patient. The TIUs software processes free-text clinical notes so they can be saved in the Veterans Health Information Systems and Technology Architecture files. We also obtained structured data from the Pharmacy Benefit Management (PBM) software to determine each patient’s medications, and International Classification of Diseases, 9^th^Revision, Clinical Modification (ICD-9-CM) codes, and laboratory data to identify reasons the patient was not prescribed medications (reasons no medications; RNM) for each patient in the cohort. Acquiring these data allowed for comparison of the concepts found in free text through NLP with VA structured data for determination of each patient’s medications or RNM.

### Sampling Strategy for Natural Language Processing Development

We used a power analysis that accounted for differences in the prevalence of clinical concepts within notes across the medical centers. We selected the sample size that involved the largest number of patients to determine the test set, in order to accommodate the rarest event (contraindications to ACEIs and ARBs) which was estimated to be 14.9% based on the HF literature [34]. We determined a sample size of 769 patients for the test set for system performance evaluation, and the remaining patients in the EPRP abstraction set (n=314) served as a separate set for training the NLP system.

#### Reference Standard Development for Natural Language Processing Development

We used Knowtator Protégé plug-in software [35] to annotate the training and evaluation (test) sets, to create a Reference Standard (RS) to undertake an accurate performance evaluation [36] of the NLP system at both the concept (eg, EF, medications, RNM) and patient (eg, overall determination or classification of a patient meeting the equivalent of CHI19) levels. We developed annotation guidelines that provided explicit examples of concepts (data) to be identified, which documents were preferred for each concept (eg, most recent echocardiogram for EF, and discharge medication reconciliation form for ACEIs and ARBs), annotation at the document level, and how to use the document-level annotations to determine the patient classification with resulting patient-level annotation [37]. We annotated 100% of the unique patients in our cohort for NLP training and testing. Two annotators independently reviewed the text documents. We measured percent agreement between the annotators across all concepts. The patient- and document-level annotations, as well as differences between concept-level annotations, were resolved via consensus determination by the two annotators with assistance from a subject matter expert (SME) cardiologist who was part of the study team. The annotators were required to achieve 90% interannotator agreement (IAA) at the concept level, and were assessed for accuracy before annotating the RS. A cardiologist (SME) reviewed and adjudicated differences when needed. We created the final RS after all differences were resolved. All cases were successfully classified by the annotators with cardiology oversight.

We used two software tools to assist annotators by preannotating concepts for subsequent verification. The first software tool, based on the Apache Unstructured Information Management Architecture (UIMA) framework [38,39], was designated *Capture with UIMA of Needed Data using Regular Expressions for Ejection Fraction* [40] and used to preannotate EF information. The second tool, the Extensible Human Oracle Suite of Tools [41], was used to preannotate ACEI/ARB medications. Preannotated concepts were read into the Knowtator software for annotators to review and finalize. Annotators reviewed preannotations as well as all other information in the document, based on the annotation guidelines.

### Natural Language Processing System Development for Information Extraction

**Figure 1:**
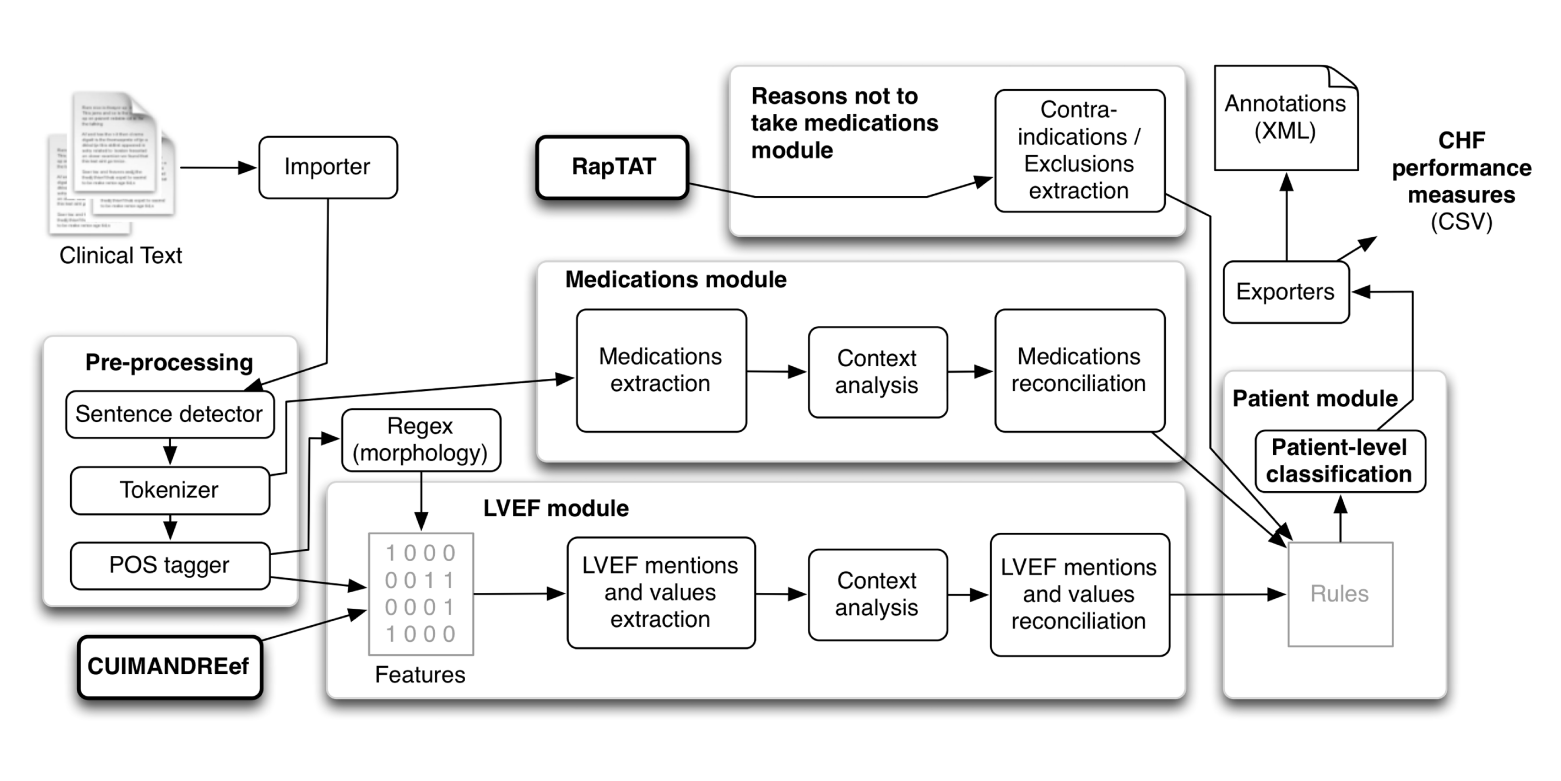
Congestive heart failure information extraction framework (CHIEF).

We based target concepts for NLP development on clinical guidelines, VA policy, and what was currently collected manually through the EPRP process [6,31]. These target concepts also served as elements in an algorithm for calculating VA CHI19 at the time of discharge. We developed an application called the Congestive Heart Failure Information Extraction Framework (CHIEF) [42-44], based on the Apache UIMA framework, to provide robustness and scalability [38]. As depicted in Figure 1, the CHIEF includes modules for (1) clinical text preprocessing (eg, detecting sentences and tokens as well as conducting syntactic analyses), (2) extracting mentions of EF as well as quantitative values, and (3) extracting mentions of medications (eg, ACEIs and ARBs). RNM were extracted with another NLP application called RapTAT [45], and the resulting data were integrated into the CHIEF.

**Figure 2:**
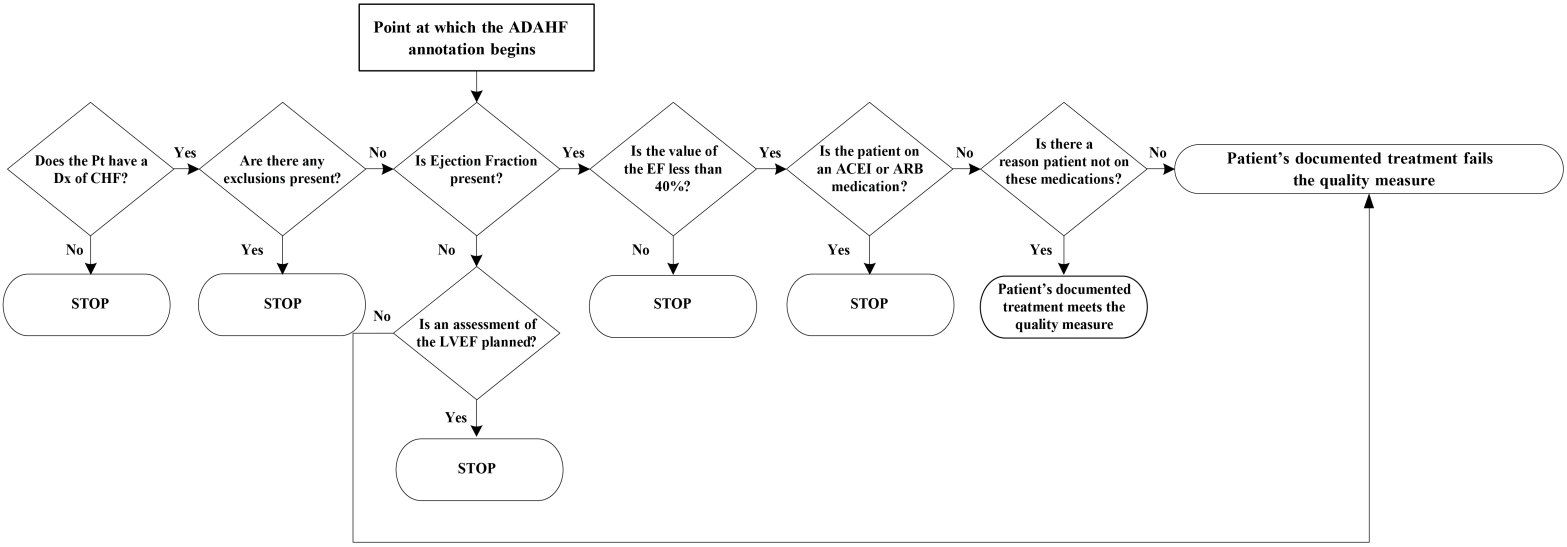
Algorithm to classify the patient as meeting the measure.

Finally, all extracted information at the concept and patient levels was compared and combined using a set of rules to classify HF treatment performance measures automatically for each patient (see Figure 2). For example, the NLP system assessed whether the patient had an EF recorded (and if the answer was yes, was it <40%?). If the EF was <40%, then the system determined if the patient was on an ACEI or ARB. If the patient was not, then the system determined if there were RNM. The patient met the measure: if the EF was present but not <40%; if the EF was <40% and there was an active prescription for an ACEI or ARB; or if the patient had an EF <40%, was not on an ACEI or ARB, and had RNM (see Figure 2).

### Key Informant and Subject Matter Expert Interviews

To inform the design of our automated system and to facilitate adoption, we interviewed both key informants and SMEs. We conducted 15 interviews consisting of four key informants and a convenience sample of 11 additional SMEs. The four key informants that were interviewed were VA quality measurement experts with national roles and VA-wide knowledge about inpatient HF quality measurements and the use of quality measurements for HF in the VA. Based on a snowball sampling design, the key informants recommended the inclusion of 11 additional VA-based SMEs. We recruited and interviewed these SMEs, who were responsible for receiving and interpreting quality monitoring data, and included VA cardiologists and HF quality experts with extensive experience in making decisions regarding the quality measures to be used and presentation of the results of quality assessments. The key informants’ and SMEs’ experience in the VA ranged from 2 to 35 years, and from 2 to 33 years in quality management.

To develop our interview questions, we drew upon the Promoting Action on Research Implementation in Health Sciences (PARiHS) framework [46,47], which postulates that evidence, context, and facilitation are central to implementation. We complemented the PARiHS framework with the Socio-Technical Model of Health Information Technology to focus on the information technology context of potential implementation [48]. We studied the potential of integrating an automated quality measurement system in the VA through these interviews, and will detail our applied thematic analysis in a future manuscript.

### Measurements

We compared the CHIEF system output to the human annotator-created RS to compute performance at the concept level and for the patient-level binary classification of meeting or not meeting the CHI19 measure. We calculated sensitivity (SN), specificity (SP), and positive predictive value (PPV) in addition to the F-measure, which is the harmonic mean of the SN and PPV [49]. We also computed the SN of the NLP test set based on the results of the EPRP review at the patient level for target concepts, and the overall binary classification of meeting the CHI19 measure. We computed Cohen’s kappa [50] parameter to determine concordance between the structured prescription data from the PBM package to determine patient medications, and both the human-annotated RS and the NLP output. Similarly, we compared ICD-9-CM codes and laboratory results to both the human-annotated RS and the NLP output to find RNM. We then summarized the interview findings to complement the system development.

### Institutional Review Board Approval

This study was approved by the University of Utah and the Tennessee Valley Healthcare System Institutional Review Boards (IRBs). Informed patient consent was waived for text document use. The IRB approved informed consent with a waiver of documentation of consent for the key informant and SME interviews.

## Results

### Documents Obtained for the Research

**Figure 3:**
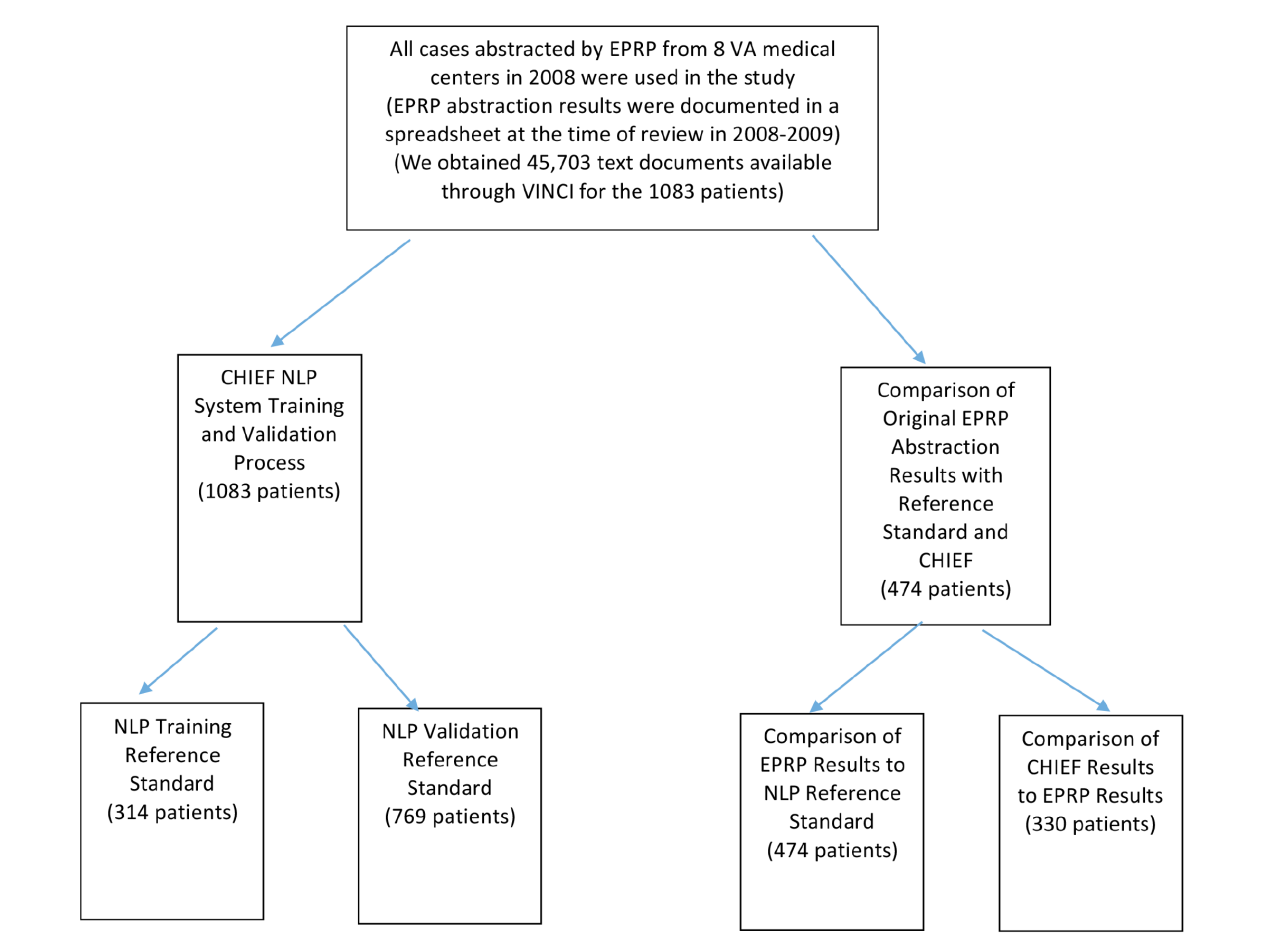
Data capture strategy.

We retrieved 45,703 free-text (TIU) documents from 1083 patients (314 in the training set and 769 in the test set). Using a systematic sample (every tenth document), we mapped the document title names to the following documents types in our corpus: history and physical, progress notes, cardiology consult, echocardiogram, pharmacy (medication reconciliation), pharmacy (other), consult (other), discharge summary, nursing note, and other (general). After mapping and during annotation, we found that EF was most commonly found in the assessment and current history sections of any note in which these sections were used (eg, history and physicals, progress notes, cardiology consults). Medications (ACEI/ARB) were most commonly found in the assessment and medication sections, LVEF was most commonly found in the echocardiogram results and assessment sections, and RNM were most commonly found in the assessment section [51]. Please see Figure 3 for the data capture strategy used in the research.

### Reference Standard Development

The IAA was found to be 91% in a pairwise comparison of all concepts within the documents in the project corpus for the RS [52]. In consultation with the cardiologist as needed, the annotation team was able to agree on this consensus RS for all patient-level classifications.

### Performance of the Natural Language Processing System

We developed the CHIEF NLP system (see Figure 1). When evaluated at the information extraction level, CHIEF extracted relevant mentions and values of LVEF, medications, and RNM with a range of *high* to *fair* recall. HF medications were extracted with recall of 97.8-99.7% and precision of 96-97.8%; mentions of LVEF were extracted with recall of 97.8-98.6% and precision of 98.6-99.4%, and RNM were less common and more difficult to extract, only reaching fair accuracy with 31.1-40.4% recall and 24.7-32.1% precision [53]. As explained earlier, this extracted information was then combined at the patient level using a set of rules. At the patient level, as shown in Table 1 and compared with the RS, CHIEF achieved almost 99% F-measure, SN, and PPV for classifying a patient admission as meeting the CHI19 measure. The SP was 85.5%. The CHIEF could also classify whether the performance measure was met, assess LVEF, and determine whether the EF was below 40%, with F-measures of 98.80%, 99.52%, and 95.52%, respectively. However, the system identified more false positives in medication prescriptions (PPV, <90%; F-measure, 93.53%). For concept extraction, we used machine learning-based approaches (fully automated) rather than rule-based or just keyword searching, and for classification we used several rules (as depicted in Figure 2). Note that for classification we programmed three sets of rules based on decisions depicted in Figure 2. The full set of rules can be found in Multimedia Appendix 1.

The lowest performance of the NLP system was measured for RNM (ie, ACEIs or ARBs), with an SN of 26.9%, SP of 99.4%, and PPV of 90.7%. This performance level was affected by RNM being the least structured, most varied, and least common of all of the concepts evaluated, with only 145 patients in our testing corpus having RNM. When we restricted our analysis to patients with an EF <40% (according to the RS) who were discharged without prescriptions for ACEIs or ARBs (n=70), performance increased slightly (SN, 33%; SP, 92.3%; PPV, 95%; and F-measure, 49%). However, when evaluating hospitalizations for which it was critical for the system determination for patient classification (n=37; EF <40% and discharged without prescriptions according to system output), the RS found more RNM than the NLP system found, but the SN of the system increased to 78%. The PPV was relatively unchanged at 81.8%, for an F-measure of 80%.

#### Concordance of Reference Standard and External Peer Review Program Results

Table 2 provides a comparison of the human-annotated RS and the NLP system output to the EPRP review findings. Of the 1083 patients, only 474 patient abstractions had the equivalent data elements to those we captured with CHIEF and were classified as meeting or not meeting the measure. Only 10 patients were present in the EPRP data who did not meet the CHI19 measure. Based on this finding, the SN is the only relevant metric, and with only 10 patients we could not get a precise estimate of any other metrics.

**Table 1:** Performance of the Congestive Heart Failure Information Extraction Framework (CHIEF) system for each patient compared to the reference standard established by human review (patient-level classification CHI19).

Patient-Level Classification	Sensitivity Estimate % (95% CI)	Positive Predictive Value Estimate % (95% CI)	F-measure
Measure CHI19^a^ met	98.9 (97.8, 99.5)	98.7 (97.6, 99.4)	98.8
Left Ventricular Systolic Function assessed	100.0 (99.5, 100.0)	99.0 (98.0, 99.6)	99.5
EF^b^ <40%	96.8 (94.6, 98.3)	95.1 (92.6, 97.0)	96.0
ACEI^c^ or ARB^d^	99.2 (98.1, 99.7)	88.5 (85.8, 90.8)	93.5
Reason not on medications	26.9 (20.0, 34.9)	90.7 (77.9, 97.4)	41.5

^a^CHI19: Congestive Heart Failure Inpatient Measure 19; LVEF >40 on ACEI/ARB at discharge.

^b^EF: ejection fraction.

^c^ACEI: angiotensin-converting enzyme inhibitor.

^d^ARB: angiotensin-receptor blocker.

**Table 2:** Sensitivity of patient-level classification of the reference standard and Congestive Heart Failure Information Extraction Framework (CHIEF) based on External Peer Review Program (EPRP) review.

Patient-Level Classification/Sensitivity	Number of Patients in Agreement with EPRP Review	Number of Patients with Corresponding EPRP Review	Sensitivity Estimate % (95% CI)
Classification in Reference Standard	469	474	98.95 (97.56, 99.66)
Classification from CHIEF	325	330	98.48 (96.50, 99.51)

**Table 3:** The External Peer Review Program (EPRP) quality measurement designation of patients in the training and test sets.

Measure	Data Present	Measure Met	Total n (%)	Number in Test n (%)	Number in Training n (%)
CHI10^a^	No	N/A	74 (6.83)	61 (7.93)	13 (4.14)
	Yes	No	4 (0.36)	4 (0.13)	0 (0.00)
		Yes	1005 (92.79)	704 (91.54)	301 (95.85)
CHI19^b^	No	N/A	600 (55.40)	433 (56.30)	167 (53.18)
	Yes	No	9 (0.83)	6 (0.78)	3 (0.31)
		Yes	474 (43.76)	330 (42.91)	144 (45.85)
CHI20^c^	No	N/A	768 (70.91)	546 (71.09)	222 (28.90)
	Yes	No	9 (0.83)	6 (0.78)	3 (0.96)
		Yes	306 (28.25)	217 (28.22)	89 (28.34)
No data on any measure			54 (4.99)	42 (5.46)	12 (3.82)
Total sample size			1083 (100.00)	769 (100.00)	314 (100.00)

^a^CHI10: Congestive Heart Failure Inpatient Measure 10; inpatient left ventricle function assessed at discharge.

^b^CHI19: Congestive Heart Failure Inpatient Measure 19; LVEF >40 on ACEI/ARB at discharge.

^c^CHI20: Congestive Heart Failure Inpatient Measure 20; LVEF >40 on ACEI/ARB prior to inpatient admission.

We compared the EPRP data with both our RS developed with SMEs, and with the results of CHIEF. When we compared the RS to the EPRP patient classifications using the EPRP findings as truth for patients meeting CHI19 in applicable cases in both the training and test sets (n=474), we found the SN of the RS to be 99.0%. We also compared the NLP results for hospitalizations in the NLP test set (n=330) to the hospitalizations for whom the EPRP provided results for CHI19 using the EPRP findings as truth for patients meeting CHI19 in applicable cases, and found an SN of 98.5% for the CHIEF. Human annotators classified 100% of cases as meeting or not meeting the measure. However, we found that there were no EPRP results for CHI19 for 55.4% of the patients assessed, even though other measures (such as CHI10 or CHI20) might have been completed, making these EPRP results noncomparable to our results. The CHIEF processed and classified 100% of patients in the test set, with 92.1% meeting CHI19. Meeting the measure required that the case was eligible for the performance measure and that the patient data showed that the case satisfied the performance required by CHI19 (see Table 3).

##### Concordance Between the Reference Standard, Natural Language Processing Output, and Structured Data

We found that the agreement (based on Cohen’s kappa) between the PBM data and the RS for RNM was 0.326, and the agreement between the PBM and the NLP system output was 0.221. Both results were interpreted as *fair* agreement [54]. We determined that the low kappa result was due to the PBM data not capturing the reasons why ACEI and ARB were not prescribed, as well as the text documents. When we performed the same calculations for laboratory and ICD-9-CM data for RNM, the laboratory data compared with the RS and NLP output provided kappa values of 0.2083 and 0.1373, respectively. The ICD-9-CM codes indicated only five patients with RNM and showed no agreement with the RS or NLP system output. Similar to the PBM data, clinical text documents are a better data source to capture reasons not to prescribe than laboratory results and ICD-9-CM data. A kappa statistic was calculated as an aggregate measure using the laboratory results and the ICD-9-CM codes as well, but did not differ from the kappa statistic for the laboratory results alone.

#### Summary Findings from Interviews

Key informants and SMEs provided valuable insights about the design of the CHIEF system and the related development and validation methods. The development team held regular meetings with key informants one to two times per year to review design decisions, such as the capture of concepts to approximate the data elements of the measure. For example, the quality measure assesses whether the patient had left ventricular systolic function assessed. The design team used the presence of an EF in the record of the patient to mean that left ventricular systolic function was assessed. Similarly, there are multiple mentions of the EF in a given echocardiogram report. The design team worked with SMEs to determine the most clinically relevant mention to use in the classification algorithm, and targeted the mention in the section of the report that is a narrative summary by the reviewing cardiologist to extract. Last, the key informants agreed that the research team could use a limited document set, rather that the entire medical record for a given patient discharge, to extract and classify the patient’s documentation as to whether or not the measure was met.

Interview respondents also discussed several areas related to how the automated NLP processes are potentially aligned with organizational goals and clinical needs. Respondents noted three potential benefits: (1) use of an automated quality measurement system could improve the efficiency of data capture and thus provide it more quickly; (2) an automated system that facilitates redeployment of resources to emerging areas is aligned with VA organizational goals and strategies; and (3) an NLP system and the resulting data could be used for clinical purposes, in addition to use in quality measurement.

An automated system has the potential to provide consistent data sources for measurement and new data regarding EF to the VA primary care almanac; it could also serve as a data source for primary care teams, VA dashboards, and clinical decision support (CDS). The system could also provide data organized in a summarized, longitudinal manner, and assist cohort and registry development.

The use of an automated quality measurement process for measuring HF quality appears to be aligned with VA organizational goals, could support the current VA culture of measurement and feedback, and provide needed data for accountability. An automated system could also facilitate meaningful use certification, further electronic quality measurement, and assist real-time (rather than retrospective) measurement.

Key informants and SMEs also suggested specific clinical uses for the NLP system and the resulting data, as follows: HF guideline and quality measurement training for providers, automated review and documentation of LVF, identification of patients needing transitional management and palliative care, summarization of clinical findings and treatment to assist clinician decision-making, and identification and contacting of patients with gaps in evidence-based care to aid quality improvement efforts (care coordination).

The interviews provided important information about the automated NLP system and its potential clinical uses. Further research is needed to identify potential technical and organizational barriers to the use of such an NLP system in the VA, as this would help determine the next steps in potential implementation.

## Discussion

### Principal Results

In this paper we report the formative evaluation of the use of the CHIEF system that integrates core algorithms reported previously [53], in addition to rules derived from existing HF guidelines, to generate a final CHI19 classification. The CHIEF processed 100% of the patients in the test set, with 92.1% of patients classified as meeting the CHI19 measure. Use of the CHIEF could potentially reduce or eliminate the need for routine human review of HF charts for the similar measures of CHF7 (HF-Outpatient LVF documented) and CHF14 (HF-Outpatient LVEF Less Than 40 on ACEI or ARB). CHF is a prevalent condition and CHIEF is an application that could provide an automated first review for HF patients to assess guideline-concordant care, and this data could potentially populate the existing EPRP dashboard automatically rather than through human review. During this process those patients who do not meet the measure could be identified; this would potentially allow a redeployment of human resources to evaluate why the care was not guideline-concordant and evaluate other quality of care issues. For example, more human resources could be used to assess patients who are at high risk for readmission, or who are frail and need additional care coordination.

The CHIEF also provides essential data that could be used in a dashboard to facilitate the identification of patients in a given provider’s panel who may need additional medications such as an ACEI or ARB therapy or other medications, as guidelines are updated. Although the EPRP abstractors have access to the entire medical record for each patient they review, they focus only on finding the required data elements within the measure, rather than on a broad quality review in which other quality of care issues may be found. We obtained good results with the CHIEF using a limited document set. These findings suggest that the CHIEF is highly reliable and that its use could reduce or eliminate the expense associated with human review of HF patient records.

### Limitations

There are several limitations to this work. First, it is likely that some clinical information was not documented in the patient charts and therefore could not be captured by the NLP system. However, we believe the impact of this missing information is minimal, given the importance and longstanding use of the HF quality measurement. Second, although the CHIEF performed well using VA text notes, it might not perform as well in non-VA settings. After training on new documents, we expect that it will perform similarly. Third, documents from only eight medical centers were used in this research; therefore, the CHIEF might under-perform initially when used with documents from other VA medical centers.

### Comparison with Prior Work

This work builds on prior research in which we developed a system for concept extraction using a rule-based method. In the current CHIEF system, we used machine learning-based methods (sequential tagging) [40]. Our informatics work is also complementary to other uses of the NLP system in cases of patients identified as having HF or classified as having a preserved or reduced EF [55,56], for the purposes of identifying patients for potential inclusion in research and those appropriate for treatment in primary care notes [57]. The relevance and importance of NLP tools in clinical practice are increasing. As such, testing and evaluating the implementation and deployment of NLP tools in clinical practice settings is an important next step.

Use of the CHIEF is also aligned with the current VA strategic plan for 2013-2018 that sets forth the principal that all initiatives be data-driven and evidence-based to help VA improve service delivery. The CHIEF has delivered promising results that could help achieve the goals of improving performance, advancing innovation, and increasing operational effectiveness and accountability in the VA, as well as in other health care organizations [58]. While CHIEF is not currently being implemented in the VA, we will seek potential implementation in VA and other settings.

Our work is important because some clinical information related to quality measures can only be found in text. Text data is not structured, so transformation of clinical text documents in a systematic, standardized process could result in its incorporation in a data warehouse across an enterprise, which would allow the use of the National Quality Forum information model designed for EHR-based quality measures, and facilitate the use of algorithms across institutions [59].

Due to the increasing availability of EHRs and the development of NLP techniques, many systems and techniques have been, and continue to be, developed to encode narrative data for a variety of uses such as: assessing the incidence rates of adverse events, evaluating the success of preventive interventions, benchmark performance across hospitals, determining cardiovascular risk factors, providing smoking cessation, providing real-time quality metrics for colonoscopies (in terms of identification of adenomas and sessile serrated adenomas), developing retrospective clinical data for use in cardiovascular research using NLP, and identifying ventilator-associated events (VAEs) and quality reporting and research in VAEs [60-62].

The Institute of Medicine envisioned a health care delivery system that would improve the quality of care and reduce costs. To accomplish this goal, it is important to create effective CDS delivered to clinicians through EHRs at the point of care [63]. The data captured from text, once transformed to structured data in the enterprise CDW, could be used in CDS.

Our methods complement other systems that identify hospitalized patients with HF in which machine learning approaches are used. Importantly, the complexity of implementation of these systems is well known and supports the assessment of barriers and facilitators for potential implementation [62,64]. The use of EHRs to automate publicly reported quality measures is receiving increasing attention, and is one of the promises of EHR implementation. Kaiser Permanente has fully or partly automated 6 of 13 the joint commission measure sets, resulting in an automated surgical site infection reporting process which reduced Kaiser Permanente’s manual effort by 80%, resulting is savings of US $2 million [65]. The VA could potentially realize reduced expenses associated with increased automation and decreased manual review of medical records for HF quality measurement.

The use of NLP for quality measures also adds to the capture of large amounts of clinical data from EHRs. The next step is to transform health care *big data* into actionable knowledge for quality improvement and research that helps to improve patient care, and potentially limit health care costs, with the aim of developing infrastructure with real-time data to support decision making [62-64,66,67]. The products of this NLP pipeline could potentially impact a number of clinical areas, including personalized CDS (eg, the suggestion to administer ACEIs/ARBs when inappropriately not administered), and could both facilitate appropriate care by promoting CDS use and prevent provider fatigue by reducing the incidence of false-positive notifications [53]. Our work is also in alignment with the recent description of the use of *big data analytics* in the VA, because the extracted data from our system has been scientifically evaluated for accuracy and reliability, and builds on the significant data resources in the CDW [33].

### Conclusions

The CHIEF system accurately classified patients for the CHI19 performance measure, with high SN and PPV. HF is an increasingly prevalent condition among patients within the VA. Our results demonstrate that automated methods using NLP can improve the efficiency and accuracy of data collection and facilitate more complete and timely data capture at the time of discharge, at a potentially reduced cost. These tools also have applications in clinical care delivery and are aligned with US national strategic initiatives to use EHR data for quality improvement.

## Appendix

Multimedia Appendix 1 Three sets of rules to classify the patient.

## Abbreviations

ACEI: angiotensin-converting enzyme inhibitor
ARB: angiotensin-receptor blocker
CHF7: HF-Congestive Heart Failure Outpatient Measure 7
CHF14: Congestive Heart Failure Outpatient Measure 14
CHI10: Congestive Heart Failure Inpatient Measure 10
CHI19: Congestive Heart Failure Inpatient Measure 19
CHI20: Congestive Heart Failure Inpatient Measure 20
CDS: clinical decision support
CHIEF: Congestive Heart Failure Information Extraction Framework
CDW: Corporate Data Warehouse
EF: ejection fraction
EHR: electronic health record
EPRP: External Peer Review Program
HF: heart failure
IAA: interannotator agreement
ICD-9-CM: International Classification of Diseases, 9th Revision, Clinical Modification
LVEF: left ventricular ejection fraction
LVF: left ventricular failure
NLP: natural language processing
PBM: Pharmacy Benefit Management
PPV: positive predictive value
PARiHS: Promoting Action on Research Implementation in Health Sciences
RNM: reasons no medications
RS: Reference Standard
SN: sensitivity
SP: specificity
SME: subject matter expert
TIU: text integration utilities
UIMA: Unstructured Information Management Architecture
VA: United States Department of Veterans Affairs
VAE: ventilator-associated event
