# Supplementary material for: Automating Quality Measures for Heart Failure Using Natural Language Processing: A Descriptive Study in the Department of Veterans Affairs
Source: JMIR Med Inform. 2018 Jan 15;6(1):e5. doi: 10.2196/medinform.9150 (PMC5789165; doi:10.2196/medinform.9150)
Supplement: Multimedia Appendix 1 [file medinform_v6i1e5_app1.pdf]

Multimedia Appendix 1

Three Sets of Rules to Classify the patient having met or not met the measure are described as follows:

Set #1

For EF < 40%,

- If EF < 40% in the most current ECHO report

- If EF < 40% in the most current cardiology consult, when there is no ECHO report is present

- If EF < 40% in the most current document that has an EF value, when no ECHO or cardiology consult is present

Set #2

For Patient on ACEI or ARB when EF < 40%

- If the patient on ACEI or ARB in the most current discharge summary

- If the patient on ACEI or ARB in the most current pharmacy reconciliation report, when there is no discharge summary

- If the patient on ACEI or ARB in the most current document that has ACEI or ARB, when there is no discharge summary or pharmacy reconciliation report

Set #3

For Reason no med when the patient is not on an ACEI or ARB medication,

- If the patient has any Reason no med in any document
